# Exterior Use of Nitrate of Silver in Conjunctivitis

**Published:** 1847-12

**Authors:** 


### *Exterior use of Nitrate of Silver in Conjunctivitis.*

To the Editor of the Annalist :

Dear Sir :—Dr. Hocken, of London, presented to the profession, some time since, through the *Lancet*, an article upon strumous conjunctivitis, and a method of cure with nitrate of silver, applied to the outside of the lids. As I had seen a good deal of the suffering attendant upon the usual application of this remedy, and its doubtful efficacy, this new plan struck me as, on one account at least, preferable to the old. I therefore determined to try it, and have done so, in a variety of cases, not merely of struma, but of acute and chronic inflammation from other causes, even in cases of granular lids, and always with the most astonishing success. I cannot recall a single instance where more benefit has not followed than I had been accustomed to expect from the old method. In some cases the intolerance of light, scalding tears, pain, and injection of the conjunctiva have been removed by one application, and the eye almost perfectly restored in twenty-four hours after; and this without the production of the least pain. It is no small advantage of this mode of using nitrate of silver, that no harm can result to the eye by its injudicious use, as may follow the introduction of the caustic into the eye, either in the solid or fluid state.

The manner of using it is to draw a clean stick of pure caustic, previously moistened, once or twice over the lids, keeping them, meantime, a little tense ; and repeating this, if necessary, in a few days. Yours respectfully JOHN S. CAMERON, M. D.

No. 120 SPRING ST., SEPTEMBER 27, 1847.

